# Supplementary material for: Target Score—A Proteomics Data Selection Tool Applied to Esophageal Cancer Identifies GLUT1-Sialyl Tn Glycoforms as Biomarkers of Cancer Aggressiveness
Source: Int J Mol Sci. 2021 Feb 7;22(4):1664. doi: 10.3390/ijms22041664 (PMC7915893; doi:10.3390/ijms22041664)
Supplement: Supplementary file 1 [file ijms-22-01664-s001.zip › Supplementary-Information_2021.docx]

**Target Score: a proteomics data selection tool applied to esophageal cancer identifies GLUT1-Sialyl Tn glycoforms as biomarkers of cancer aggressiveness**

Sofia Cotton^1,2,3,4^, Dylan Ferreira^1,2,3,4^, Janine Soares^1,2,5^, Andreia Peixoto^1,3,4^, Marta Relvas-Santos^1,2,3,4,6^, Rita Azevedo^7^, Paulina Piairo^8^, Lorena Diéguez^8^, Carlos Palmeira^1,9,10^, Luís Lima^1,11^, André M.N. Silva^6^, Lúcio Lara Santos^1,2,10,11,12^, José Alexandre Ferreira^1,2,11^

^1^Experimental Pathology and Therapeutics Group, IPO Porto Research Center (CI-IPOP), Portuguese Oncology Institute (IPO Porto), 4200-072 Porto, Portugal; ^2^Institute of Biomedical Sciences Abel Salazar (ICBAS), University of Porto, 4050-313 Porto, Portugal; ^3^Institute for Research and Innovation in Health (i3S), University of Porto, 4200-135 Porto, Portugal; ^4^Institute for Biomedical Engineering (INEB), Porto, Portugal, 4200-135 Porto, Portugal; ^5^QOPNA/LAQV, Department of Chemistry, University of Aveiro, Campus Universitário de Santiago, 3810-193, Aveiro, Portugal; ^6^REQUIMTE-LAQV, Department of Chemistry and Biochemistry, Faculty of Sciences of the University of Porto, 4169-007 Porto, Portugal; ^7^Institute of Biomedicine, University of Turku, FI-20014 Turku, Finland; ^8^Medical Devices Research Group, International Iberian Nanotechnology Laboratory (INL), 4715-330 Braga, Portugal; ^9^Departament of Immunology, Portuguese Institute of Oncology of Porto, 4200-072 Porto, Portugal; ^10^Health School of Fernando Pessoa University, 4249-004 Porto, Portugal; ^11^Porto Comprehensive Cancer Center (P.ccc), 4200-072 Porto, Portugal; ^12^Department of Surgical Oncology, Portuguese Institute of Oncology, 4200-072 Porto, Portugal;

**Corresponding author:**

José Alexandre Ferreira (jose.a.ferreira@ipoporto.min-saude.pt)

Experimental Pathology and Therapeutics Group, Research Centre, Portuguese Oncology Institute of Porto, R. Dr. António Bernardino de Almeida 62, 4200-072 Porto, Portugal; Tel. +351 225084000 (ext. 5111).

**Running head:** Targetable glycoproteins in esophageal cancer

**Keywords:** cancer biomarkers; circulating tumours cells; esophageal cancer; glycomics; glycoproteomics; bioinformatics

**Supporting Material and Methods**

Proteomics data generates a high number of protein identifications, warranting tools that allow to easily pinpoint biomarkers of potential clinical interest. The Target Score algorithm was developed to provide an easy and comprehensive navigation throughout pre-existing molecular databases, enabling to extract information that maybe used to rank identified biomarkers in relation to prognosis and potential targetability. It was designed to attribute higher scores to glycoproteins overexpressed in cancer, located at the cell membrane, and associated with poor prognosis, while penalizing glycoprotein remaining at the same subcellular location than previously observed in healthy tissues. As primary source of information for this study, we used the Protein Atlas database (https://www.proteinatlas.org/) consulted in March 2020. Nevertheless, it sets a roadmap that maybe used to navigate through other databases containing information on protein expression in cancer and healthy tissues, their subcellular location and cancer prognosis. Accordingly, the score system results from the sum of the following nine variables: i) location in healthy cells (membrane: 0 points; other subcellular locations: 1 point); ii) location in cancer cells (membrane: 1 point; cytoplasm and/or other subcellular locations: 0 points); iii) expression in healthy esophageal epithelium at the plasma membrane (negative: 3 points; low: 2 points; moderate: 1 point; high: 0 points); iv) expression in head-and-neck cancer (negative: 0 points; low: 1 point; moderate: 2 points; high: 3 points); v) prognosis value in cancer (high expression is not prognostic and/or associates with favorable prognosis: 0 points; high expression associates with poor prognosis: 1 point); vi) expression in lymphoid tissues at the plasma membrane (not expressed: 1 point; expressed: 0 points); vii) expression in gametes (not expressed: 1 point; expressed: 0 points); viii) index of expression in healthy tissues at the plasma membrane (varies from 3 points (no expression) to 0 (high expression)).


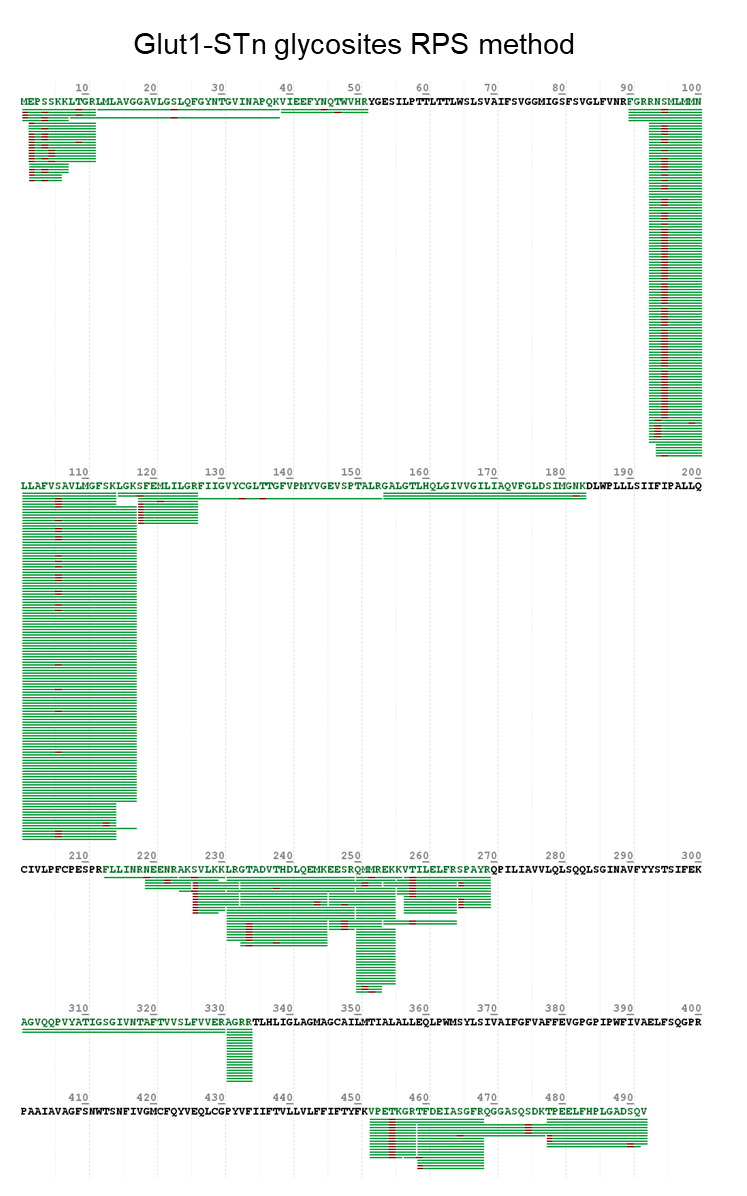


**Figure S1. GLUT1-STn glycopeptides (green) and glycosites (red) identified in ESCC using the RPS method.** Assignments were made using the Byonic software.

**
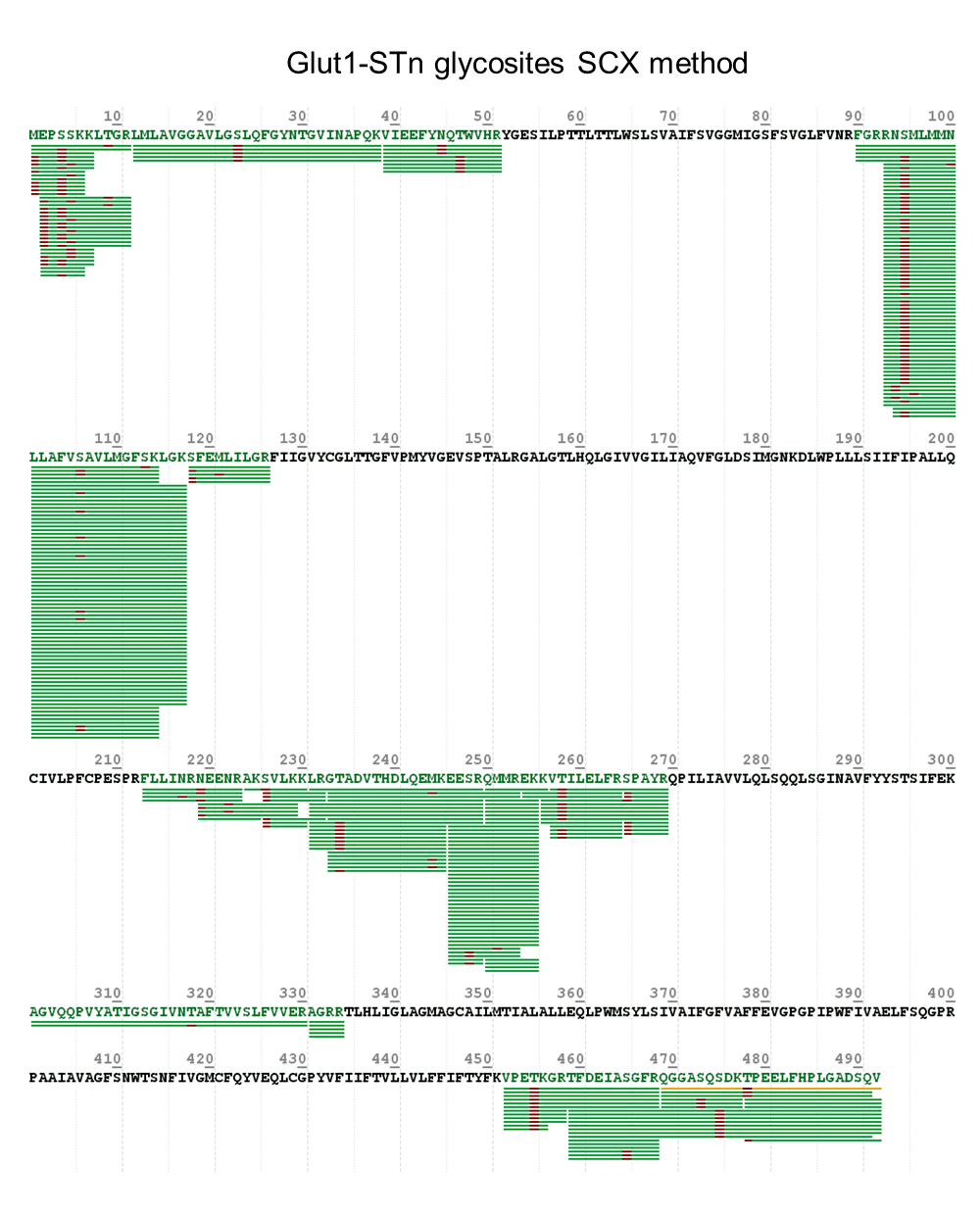
**

**Figure S2. GLUT1-STn glycopeptides (green) and glycosites (red) identified in ESCC using the SCX method.** Assignments were made using the Byonic software.
